# Supplementary material for: Mechanism of allosteric activation of human mRNA cap methyltransferase (RNMT) by RAM: insights from accelerated molecular dynamics simulations
Source: Nucleic Acids Res. 2019 Jul 22;47(16):8675–92. doi: 10.1093/nar/gkz613 (PMC7145595; doi:10.1093/nar/gkz613)
Supplement: gkz613_Supplemental_Files [file gkz613_supplemental_files.zip › Suppl_material.pdf]

SUPPLEMENTARY FIGURES AND TABLES

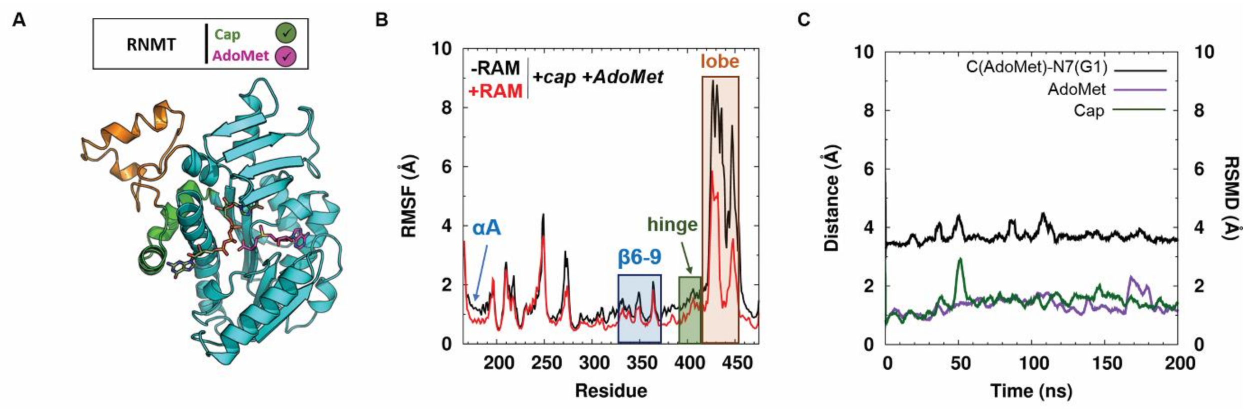

Supplementary Figure S1. RNMT modelled with the bound cap and AdoMet in the absence of RAM.

(A) Representative snapshot of RNMT in complex with the cap (green) and AdoMet (magenta) in a methyl-transfer reactive conformation.

(B) RMSF values (in Å) of the RNMT obtained from the aMD simulation.

(C) Time-evolution of the distance between AdoMet (CH3) and the cap G_0 (N7 atom) (black line). The RMSD of AdoMet (magenta) and cap (green) are plotted against the secondary (right) vertical axis.

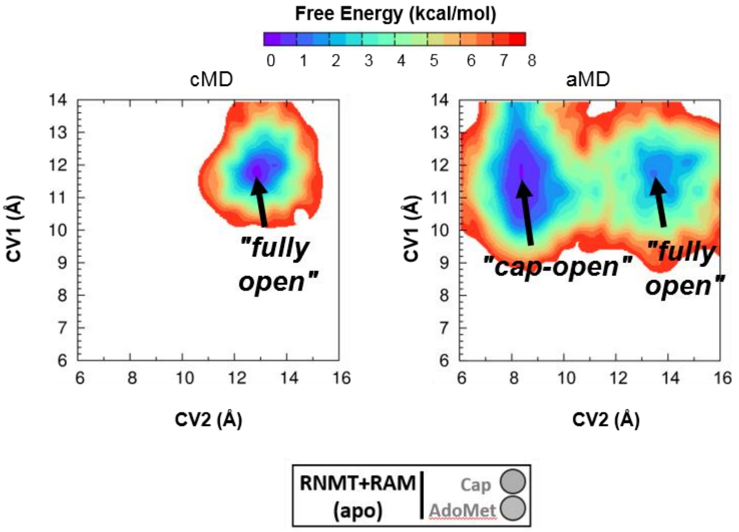

Supplementary Figure S2. Illustrating sampling of the conformational space in standard and accelerated MD. Conformational free energy landscape of the active site of RNMT as obtained in the 200 ns cMD (left) and 200 ns aMD (right) simulation of RNMT-RAM.

To ensure a fair comparison cMD simulation of this system was extended from 100 to 200 ns, i.e. the same length as in aMD simulation

System	E_p	α_p	E_D	α_D
**RNMT**	-214,473	15,272	5,238	248
**RNMT+RAM**	-218,633	15,175	5,985	284
**RNMT (+AdoMet +Cap)**	-180,810	12,553	5,361	250
**RNMT+RAM (+AdoMet +Cap)**	-149,123	10,439	6115	285

Supplementary Table S1. aMD parameters used in the present simulations. The choice of the parameters was initially based on the guidelines from previous works (43-45). Further adjustments were done using the preliminary aMD simulations. Values of all the parameters are given in kcal mol^{-1}.

Interaction		Distance (Å)	
**RNMT**	**Cap**	**cMD**	**aMD**
N176 (ND2)	(O3')	4.0 (0.5)	4.3 (1.0)
H288(ND2)	(O6)	3.9 (0.7)	4.1 (1.2)
Y289 (OH)	(O6)	2.9 (0.4)	3.7 (1.5)
E370 (OE2)	(N1)	2.8 (0.1)	2.9 (0.4)
E370 (OE1)	(N2)	3.0 (0.2)	3.0 (0.4)
Y467 (OH)	(N3)	3.1 (0.4)	4.2 (1.4)
Y467 (OH)	(O2')	3.3 (0.5)	3.6 (0.9)

Interaction		Distance (Å)	
**RNMT**	**AdoMet**	**cMD**	**aMD**
K180 (NZ)	(COO^-)	2.8 (0.1)	2.9 (0.3)
G205 (CO)	(NH_3^+)	2.8 (0.1)	2.8 (0.2)
D227 (OD1)	(O2/O3)	2.5 (0.1)	2.6 (0.1)
D261 (OD1)	(NH2)	2.8 (0.1)	2.8 (0.1)
S262 (NH)	(N1)	3.2 (0.2)	3.2 (0.2)
Q284 (CO)	(NH_3^+)	2.8 (0.1)	2.9 (0.3)

Supplementary Table S2. Key RNMT-cap and RNMT-AdoMet hydrogen-bonding and salt-bridge interactions observed in the simulations. The average distances for the key interactions computed over the cMD and aMD trajectories are given in Å, and the standard errors are reported in parentheses. Atoms between which the distance is measured are listed in the left two columns.

Interaction		Distance (Å)		
RAM	RNMT	Crystal structure	cMD	aMD
R18 (NH)	Y351 (O)	2.6	2.7 (0.1)	2.9 (0.3)
D23 (OD)	Y374 (OH)	3.2	4.5 (0.9)	3.3 (1.0)
Y29 (OH]	E323 (OE)	2.4	2.8 (0.3)	3.4 (1.2)
Y29 (OH)	N320 (ND)	3.2	3.8 (0.6)	3.8 (0.9)

Supplementary Table S3. H-bonding interactions between RAM and the 315-375 region of RNMT. The distances in the crystal structure and average distances computed over the cMD and aMD trajectories are given in Å, and the standard errors are reported in parentheses.
